# Recombinant gas vesicles from *Halobacterium sp*. displaying SIV peptides demonstrate biotechnology potential as a pathogen peptide delivery vehicle

**DOI:** 10.1186/1472-6750-8-9

**Published:** 2008-01-31

**Authors:** Marinko Sremac, Elizabeth S Stuart

**Affiliations:** 1 Department of Plant, Soil and Insect Sciences, University of Massachusetts, MA 01003, USA; 2 Department of Microbiology, University of Massachusetts, Amherst, MA 01003, USA

## Abstract

**Background:**

Previous studies indicated that recombinant gas vesicles (r-GV) from a mutant strain of *Halobacterium sp*. NRC-1 could express a cassette containing test sequences of SIVmac *gag *derived DNA, and function as an antigen display/delivery system. Tests using mice indicated that the humoral immune response to the *gag *encoded sequences evoked immunologic memory in the absence of an exogenous adjuvant.

**Results:**

The goal of this research was to extend this demonstration to diverse gene sequences by testing recombinant gas vesicles displaying peptides encoded by different SIV genes (SIV^*tat*, *rev *or *nef*^). Verification that different peptides can be successfully incorporated into the GvpC surface protein of gas vesicle would support a more general biotechnology application of this potential display/delivery system. Selected SIVsm-GvpC fusion peptides were generated by creating and expressing fusion genes, then assessing the resulting recombinant gas vesicles for SIV peptide specific antigenic and immunogenic capabilities. Results from these analyses support three conclusions: (i) Different recombinant *gvpC*-SIV genes will support the biosynthesis of chimeric, GvpC fusion proteins which are incorporated into the gas vesicles and generate functional organelles. (ii) Monkey antibody elicited by *in vivo *infection with SHIV recognizes these expressed SIV sequences in the fusion proteins encoded by the *gvpC*-SIV fusion genes as SIV peptides. (iii) Test of antiserum elicited by immunizing mice with recombinant gas vesicles demonstrated notable and long term antibody titers. The observed level of humoral responses, and the maintenance of elevated responses to, Tat, Rev and Nef1 encoded peptides carried by the respective r-GV, are consistent with the suggestion that *in vivo *there may be a natural and slow release of epitope over time.

**Conclusion:**

The findings therefore suggest that in addition to providing information about these specific inserts, r-GV displaying peptide inserts from other relevant pathogens could have significant biotechnological potential for display and delivery, or serve as a cost effective initial screen of pathogen derived peptides naturally expressed during infections *in vivo*.

## Background

The goal of this research was to generate and test an innovative, cost effective antigen display and delivery system that uses micro particulate gas vesicles (GV). Although it has been long known that portions (epitopes) of molecules can be recognized by the immune system and the antibodies stimulated against the correct epitope(s) can elicit protective immunity, recently there has been significantly increased interest in the exploration of novel approaches to vaccine development and immunogen delivery. Thus growth of biotechnology and development of new tools now support the important expansion to include a diversity of new aspects that support moving beyond conventional immunization approaches and obtaining new information defining vaccine components [1-3].

*Halobacterium sp*. NRC-1 is a remarkable organism that exhibits a number of characteristics that are significant to its biotechnology utility. Culturing is simple, DNA mediated transformation may be accomplished at high efficiency and proteins and organelles can be released from the halobacteria by simple lysis in hypotonic medium. The *Halobacterium sp*. NRC-1 genome is completely sequenced [4] and the genomic data supports a wide range of studies that enhance its potential for commercial uses, as well as basic biochemical analyses of adaptation to extreme conditions. In addition, both an halobacterium transformation system [5,6] and *E. coli/Halobacterium sp*. shuttle plasmids, developed for basic genetic studies, are directly applicable to biotechnological applications. The cell envelope of *Halobacterium salinarum *consists of a single lipid bilayer membrane surrounded by an S-layer assembled from the cell-surface glycoprotein. This fact is important in terms of simple release of gas vesicles, or cytosolic proteins, from the organism. The gas vesicles themselves are unique organelles naturally produced by halophilic archaea and their biogenesis is inherently quality controlled by the organism. Fourteen *gvp *genes have been identified as encoding components involved in the genesis of organelles, and the regulation of *gvp *gene expression has been shown to occur at the transcriptional and translational levels [7-10].

Gas vesicle organelles are cylindrical-shaped particles with conical ends about 200 nm long, and composed of an inflexible, thin (20Å) proteinaceous (lipid free) membrane [11-13] that encloses a gas-filled space. The lipid free membranes form a barrier to liquids but are permeable to many dissolved gases such as nitrogen, oxygen, carbon dioxide and methane. The membrane has an extremely stable, two-dimensional crystalline lattice composed of two major proteins, GvpA and GvpC. The GvpA protein is highly conserved and forms a linear crystalline array of ribs that form the cylindrical shell and conical ends of the gas vesicle. GvpC is located on the vesicle outer surfaces and adds strength, stability and shape. Previous findings suggested that GvpC is present on the external surface of the vesicle and functions as a "molecular glue" to enhance the membrane stability [14]. With regard to peptide display, these characteristics are desirable in terms of antigen presentation and are potentially important for applications involving epitope display, a key for vaccine development. In this context, previous findings indicate there is no evidence of harmful effects from consuming native halobacteria [15,16] so that recombinant gas vesicles should be suitable for use in oral as well as parenteral delivery.

Electron microscopy has demonstrated that the surface of the GV itself is highly organized suggesting it would support organized presentation of encoded antigen, and it is known that these stable protein structures themselves can provide an intrinsic adjuvant activity. Preliminary evidence demonstrated that gas vesicles can be genetically manipulated to display peptides coded by an insert of irrelevant DNA that specified a sequence of six amino acids, and peptide immunogenecity and antigenicity were verified both *in vitro *and *in vivo *[17,18]. Initial studies with the *gag *gene from SIVmac have raised the possible utility as a display/delivery system using gas vesicles to present pathogen peptide sequences up to 235 amino acids; these results also suggested the recombinant organelles were intrinsically self-adjuvanting [18,19]. Thus the preceding studies indicated the GvpC protein is sufficiently flexible to tolerate exogenous sequence inserts and thus support the principle of gas vesicles as a recombinant component for pathogen display/delivery. The studies presented below tested the generalized applicability of this technology using selected DNA segments encoding pathogen peptide sequences of diverse size and native gene function. These were assessed for plasmid retention of the exogenous DNA sequences, gas vesicle retention of the recombinant protein and the long term maintenance of elicited humoral responses to the exogenous peptide sequences displayed by the r-GvpC protein that had been incorporated into the organelle.

## Results

Our earlier studies to generate recombinant gas vesicles provided evidence that when inserted into an appropriate site in the *gvpC *gene, this halobacterial gene would accept SIV *gag *gene DNA segments of different lengths and the resultant sub microscopic organelles would express this recombinant protein at the gas vesicle surface [19]. To demonstrate the potential flexibility and gene independent nature of surface expression using this cassette based system, we applied the methods outlined below and prepared a series of 21 different plasmids that encompassed the entire SIV genome. The three gene segments selected for detailed study were used to transform the Vac^- ^mutant *Halobacterium sp*. NRC-1, strain SD109 to obtain recombinant gas vesicles. After expanding the different initial transformant populations, the functional expression of the three fragments, each representing a different insert length and a different gene, was then tested through assessments of the gas vesicles produced. Functional (floating) recombinant gas vesicles were readily isolated from each of the transformants and then tested to detect recombinant GvpC expressing the SIV^Tat, Rev or Nef1 ^encoded peptide and assessed for recognition as SIV genome derived using sera from an SHIV infected monkey. They then were used to immunize mice in order to test for their capacity to stimulate peptide specific humoral response in the absence of exogenous adjuvant and for the retention of titers for an extended time period.

### Generation of DNA components

**Figure 1:**
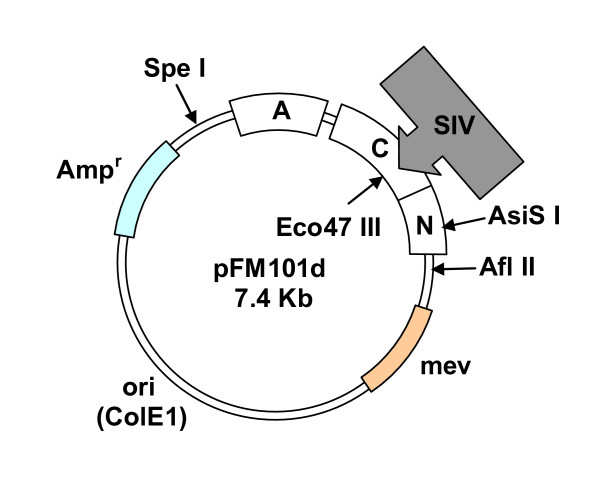
**Strategy for incorporating SIV DNA sequences into the *gvpC *gene carried in plasmid pFM101d**. Plasmid pFM101d contains the rightward gas vesicles operon with the *gvp *genes (A, C and N). A unique Eco47 III site at location "d", within the *gvpC *gene, allowed insertion of the exogenous DNA fragments. The unique restriction enzyme sites Spe I, AsiS I and Afl II allowed manipulation/isolation of this cluster of gas vesicle genes by appropriate restriction digests. The antibiotic resistance genes are indicated with the boxes and allow selection of transformed *E. coli *or SD109 using Ampicilin (Amp) and Mevinolin (mev), respectively.

The 21 segments constituting the SIV genome were successfully amplified previously and for larger genes, e.g. *Nef*, the multiple fragments of the same gene were numbered numerically starting at the amino terminal end. Table 1 shows the PCR primers used to amplify the specific gene segments studied in detail here as well as the primers for the 350 bp internal segment amplified from the *gvpC *gene. The individual SIV gene segments were then expanded, using pUC19 and transformation of *E. coli *DH5α, followed by cloning into a second vector pFM101d shown in Figure 1. Following the steps outlined in the Methods section, the selected blunt end SIV fragments were then ligated into an Eco47 III site in the *gvpC *gene at the position designated as "d" in the rightward operon in the plasmid pFM101d and used for the transformation of *E. coli*. After expanding *E. coli *transformants, rightward operons with their SIV derived DNA insert were harvested from the plasmids using the Spe I and AsiS I. Each recombinant operon was then ligated into plasmid pMS104d using the methods described previously for pFM104d [19].

**Table 1:** Primers sequences for PCR amplification of DNA encoding the proteins examined here.

**Fragments**	**Primers**	**Sequence (5' to 3')**	**Size (bp)**
**Tat**	Tat F	ggg atg gag aca ccc ttg aag	150
	Tat R	ggg atg ata gca aca cct ctt	
**Rev**	Rev F	ggg tca gca gat ccc tat cca	243
	Rev R	ggg cgg act ctt tgc aac gtc	
**Nef1**	Nef1 F	ggg atg ggt ggc gct att tcc	642
	Nef1 R	ggg ctt cca tgc cag tac ctc	
**gvpC**	gvpC F	ctc ctg ctg tga ttc tgc ga	1,070
	gvpC R	cat cct cac cgt act cgt cg	
	gvpC – Kpn I	cta tgg cca cga gat cac g	350

DNA sequences required to generate recombinant GvpC and demonstrate the successful insertion and retention of test SIV DNA sequences utilized PCR amplification and the forward (F) and reverse(R) primers listed above.

**Figure 2:**
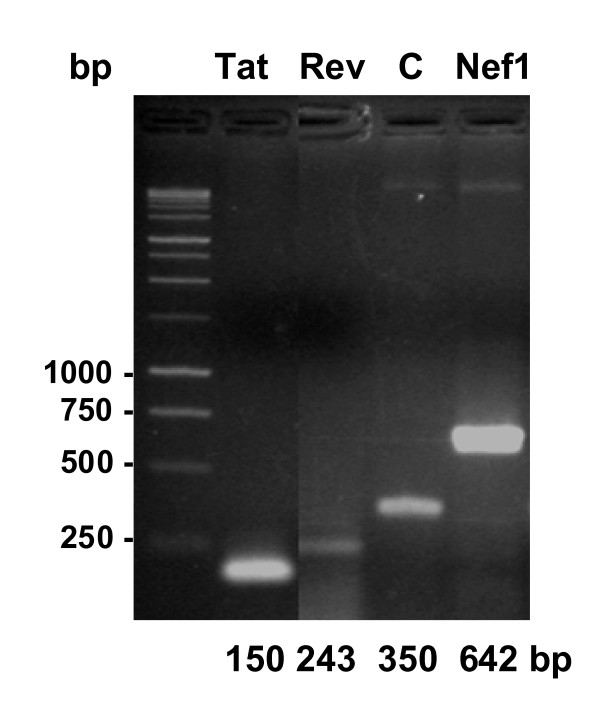
**Verification of SIV DNA sequence retention in the *gvpC *gene following plasmid amplification**. Following expansion of the pFM101D-SIV *tat, rev *and *nef1 *plasmid populations, DNA was tested for retention of the SIV specific insert carried by the recombinant *gvpC *genes. The agarose gel shows the PCR amplicon sizes of the selected SIV gene fragments and are indicated along the horizontal axis: *tat*:150, *rev:*243 and *nef1*:642 bp. Their presence inherently verifies SIV DNA retention by each r-*gvpC *genes. The Control (C) is an internal fragment from the *gvpC *gene and serves as a marker for the migration of this 350 bp fragment. The relevant primers are shown in Table 1.

In order to confirm that these plasmids retained the inserted SIV DNA sequences, the DNA was isolated from each different transformant population, and from the wild type, *Halobacterium *sp. NRC-1. Using the appropriate forward and reverse primer specific for the *gvpC *gene of each of the incorporated SIV derived DNA segments and shown in Table 1, the DNA was PCR amplified. As shown by the agarose gel in Figure 2, these amplification products indeed demonstrated the expected size relative to the 1 kb standard and to the Control (C), the internal 350 bp fragment from the native *gvp*C gene of *Halobacterium *sp. NRC-1. The electrophoretic mobility of amplification products demonstrates successive increases in base pair (bp) sizes, fully consistent with the selected SIV genomic DNA inserts. As the amplicons of the DNA inserts demonstrate, each of the three different exogenous DNA sequences had been stably retained in the recombinant *gvpC *gene contained in the rightward gas vesicle operon.

### Transformation of Vac^- ^Halobacterium strain SD109

**Figure 3:**
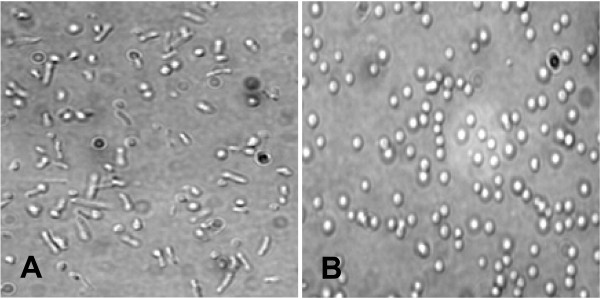
**Spheroplast formation by the gas vesicle deficient strain SD109 results in a distinctive, spherical morphology**. With the addition of spheroplasting solution and 0.5 M EDTA, the rod shape halobacteria cells (**Panel A**) rapidly changes into the transformable spheroplast form (**Panel B**) exhibiting a characteristic change in morphology detectible microscopically. Spheroplasts can then be transformed with the recombinant plasmids pMS104D::*tat*,::*rev *or *::nef1 *carrying the native leftward operon of the gas vesicle gene cluster and the rightward operon carrying an inserted SIV fragment of interest. Original magnifications 1,000 ×.

**Figure 4:**
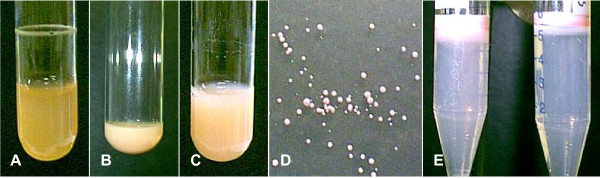
**Growth by SD109 transformants producing gas vesicles is visually distinguishable from the Vac^-^SD109 mutant**. **Panels A-E **show typical cultures of wild type or transformed halobacteria. **Panel A: **the gas vesicle deficient (GV^-^) strain SD109 which in natural color exhibits a characteristic rust brown appearance; **Panel B: **a culture of the wild type gas vesicles (GV^+^) from *Halobacterium sp*. NRC-1, exhibits the characteristic milky appearance of GV^+ ^cultures. **Panel C: **a culture of transformed halobacteria strain SD109 expressing the *nef1 *encoded peptide exhibits the characteristic rounded morphology of GV^+ ^cultures when plated on agar media with the slightly pink tinged white coloration. **Panels D **and **E: **Isolated recombinant gas vesicles, like the native ones are evident as a white ring at the interface of the air/5% NaCl solution. Flotation of the r-GV to this interface verifies the chimeric GV isolated from SD109 transformed with pMS104D::*tat *or ::*nef1 *are functional.

The Vac^- ^(minus) mutant, *Halobacterium *strain SD109 lacks the entire gas vesicle gene cluster and was used for transformation following the protocols described in the Methods. Spheroplasts were prepared from the Vac^- ^SD109 strain and can be readily distinguished from the normal rod shape of these halobacteria by microscopic examination (Figure 3A and 3B). This fact supports simple and straightforward verification that the spheroplast form required for transformation of the Vac^- ^mutant has been achieved. The spheroplasts were then transformed using pMS104D::*tat*, ::*rev *or ::*nef1 *and methods described below. The putative SD109 transformants were cultured under mevinolin selection and could be readily assessed for gas vesicle production. As seen in the comparison of liquid cultures shown in Figure 4, Vac^- ^and Vac^+ ^cultures are readily distinguishable. Figure 4A shows the slightly cloudy appearance of a Vac^- ^SD109 culture which appears brownish orange in color. In contrast, Figure 4B–C show the typical milky white culture appearance of the gas vesicle containing wild type *Halobacterium sp*. NRC-1 or successfully transformed SD109 strain. Similarly, visual inspections of transformants grown on plate media, shown in Figure 4D, also supports visual tracking for successful transformation to produce Vac^+ ^(plus) recombinants. Figure 4E shows examples of isolated r-GV and these clearly display the characteristic pink-tinged white band of gas vesicles at the interface of the 5% NaCl solution with the overlaying air; GV flotation at this interface verified that the recombinant GV are functional.

Thus the initial findings have been verified and extended to transformations using inserts of diverse DNA sizes and encoded gene function. This is a key step in r-GV production and the generation of functional product that can be readily harvested. The actual biosynthesis of the gas vesicle product and the retention of gas vesicle function, i.e. floatation of the isolated product, can be readily assessed visually. In combination, these findings demonstrate that the transformations with each of the selected recombinant plasmids (pMS104D::*tat*, ::*rev *and ::*nef1*) were successful and had conferred a functional, Vac^+ ^status to the recipient SD109 Vac^- ^mutants, irrespective of the insert selected for these studies, or its native function.

### Identification of the recombinant GV proteins by Western blot

In our earlier studies the GvpC protein was clearly evident when anti-GvpC antiserum was used. Recombinant SIVsm-GvpC protein expressing sequences of the *gag *gene was identifiable using antiserum from SIVsm challenged monkeys and standard ELISA titration of gas vesicles containing chimeric SIVsm-GvpC^Gag ^protein demonstrated the presence of recognizable SIV *gag *gene insert expressed by r-GvpC^Gag ^[19]. To demonstrate that the expression of recombinant peptide originally examined using a *gag *sequence was not insert specific or unique, this study examined recombinant SD109 transformants expression of the three different SIV encoded peptides within the framework of recombinant GvpC protein. Gas vesicles were isolated from independent, replicate cultures of each of the different recombinant populations. These were analyzed by Western blot for the presence of recombinant GvpC protein exhibiting the anticipated increase in molecular weight and recognition by the specific anti-GvpC antibody. Although halobacterial proteins behave anomalously in analyses using the standard analytical technique of SDS-PAGE [20], nevertheless the approximate molecular weight (MW) can be assessed by adding the molecular weight of the SIV insert to apparent molecular weight previously demonstrated in this system. The results are shown in Table 2 and indicate that the SDS-PAGE technique should readily detect altered electrophoretic mobility reflecting insert associated size differences. Gas vesicles putatively expressing recombinant GvpC containing Tat, Rev or Nef1 peptides were isolated from the appropriately transformed SD109 cultures. Gas vesicles similarly isolated from wild type organisms, *Halobacterium sp*. NRC-1, provided the molecular weight marker for the wild type GvpC protein used in the immuno-identifications. Using standard protocols for SDS-PAGE and then Western blots, all samples were transferred to Immobilon membranes and probed with appropriate antibodies to detect the proteins of interest.

**Table 2:** Molecular Weight and pI for the proteins examined here.

**Characteristics**	**GvpC**	**Tat**	**Rev**	**Nef1**
**pI**	3.73	4.92	8.17	5.31
**pI (Fusion proteins)**		3.83	3.88	4.07
**MW (Da)**	42,392	5,743	9,313	24,733
**Expected MW**^†^		48,117	51,687	67,106
**Predicted MW**^‡^	60,000	65,743	69,313	84,733

† Expected MW is calculated as GvpC + SIV insert; Tat, Rev and Nef1 respectively.

‡ Predicted MW is based on results for GvpC from wild type GV (NRC-1) where the protein band was indicated at ~ 60 kDa [18]. Accordingly, the MW for the recombinant proteins was calculated as a sum of the specific insert and the MW for the GvpC. Isoelectric points were calculated using the Web page for pI/MW calculation [42].

**Figure 5:**
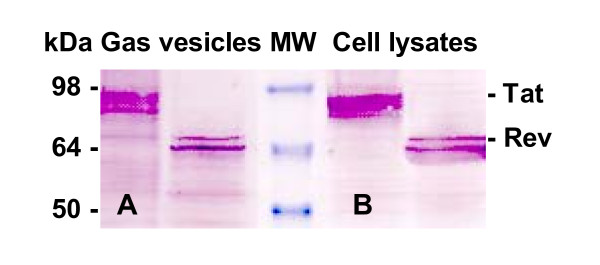
**Verification of recombinant GvpC protein formation and incorporation into functional gas vesicles**. Western blots were used to assess putative recombinant gas vesicles (**Panel A**) and cell lysates (**Panel B**) for the presence of r-GvpC protein. Here samples are resolved in 8–16% SDS-PAGE gels, immunoblotted and probed with rabbit anti-GvpC antibody. The size range of the protein standards (kDa) is indicated at the left and the recombinant GvpC protein bands are identified at the right.

**Figure 6:**
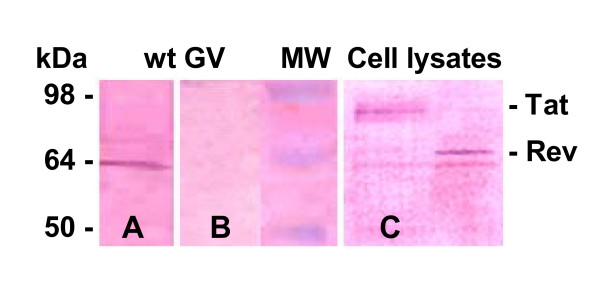
**Verification that recombinant gas vesicles have GvpC proteins containing both halobacterial and SIV encoded peptides**. Western blots of putative recombinant gas vesicle samples from cultures of SD109 transformed with pMS104D::*tat *or ::*rev *demonstrate the presence of chimeric GvpC proteins. Electroblotted wild type gas vesicles samples (wt-GV) prepared from the NRC-1 were incubated with purified rabbit anti-GvpC antibody or with monkey anti-SHIV antibody. As shown, the GvpC protein is clearly detected by anti-GvpC antibody (**Panel A**), but this protein is not detected by anti-SHIV antibody (**Panel B**). In cell lysates, Tat and Rev sequences contained in the putative recombinant GvpC protein are each detected using anti-SHIV antibody (plasma number R94085) from a monkey infected *in vivo *with SHIV virus (**Panel C**). The recombinant chimeric protein bands identified here correspond in size to bands identified with anti-GvpC antibody (see **Figure 5 Panel B**).

GvpC protein was identified by Western blot membrane incubation with anti-GvpC rabbit serum generated by immunizations using a lacZ-GvpC fusion protein and noted previously [14,19]. Gas vesicle preparations were isolated from populations transformed to incorporate Tat or Rev amino acid sequences. As Figure 5 demonstrates, the anti-GvpC antibody recognized GvpC contained in the putative fusion proteins. As this Western blot also indicates, characteristic differences in apparent molecular weight are evident for the recombinant bands recognized by the anti-GvpC antibody. These differences allow discrimination from the wild type GvpC protein (see in Figure 6, Panel A), and are consistent with an expected differentially increased molecular weight of the recombinant GvpC proteins produced by the transformants. The differences also are consistent with the anomalous behaviour of halobacterial gas vesicle proteins when analyzed by SDS-PAGE gels.

**Figure 7:**
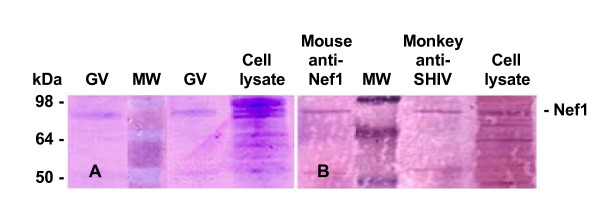
**Immunoblots verify *nef1 *DNA also is expressed by r-GvpC^Nef1 ^and carried by recombinant gas vesicles**. SDS-PAGE and Western blots demonstrate that Nef1 also is successfully expressed and can be detected in lysates and r-GV of SD109 transformed with pMS104D::*nef1*. The Coomassie Blue stained 12% SDS-PAGE gels shows resolution of proteins in gas vesicles or lysates from these transformed SD109 cultures (**Panel A**). Duplicates of these resolved proteins were electroblotted (**Panel B**) and the presence of the Nef1 peptide in chimeric r-GvpC, identified by both absorbed anti-Nef1 sera from mice immunized with r-GV^Nef1 ^and the monkey anti-SHIV antibody (plasma number R94085). Antibody binding shows recognition of Nef1 peptide within the recombinant GvpC protein. The mouse anti-Nef1 serum was used at a 1:250 dilution; the monkey anti-SHIV antibody was used at a 1:200 dilution. The standards (kDa) are shown on the left and the immuno detected band for recombinant Nef1 containing r-GvpC protein is identified on the right.

A comparison with the migration of native GvpC released from wild type gas vesicles in the lane labeled wt (NRC-1), a control included in Figure 6, shows the normal mobility of this wild type protein (Panel A). At the left side of Figure 6, Panel A shows the anti-GvpC antisera recognizes GvpC of wild type NRC-1 gas vesicles, while Panel B demonstrates that the anti-SHIV monkey sera does not recognize wild type GvpC. Panel C provides evidence that this same anti-SHIV monkey serum does recognize SIV encoded peptides within the recombinant GvpC protein (r-GvpC^Tat^, and r-GvpC^Rev^). Thus, as expected based on Table 2, the Western blots have demonstrated the expected increased apparent molecular weight of the r-GvpC proteins. More importantly, the fact that anti-SHIV monkey serum fails to recognize wild type GvpC validates this plasma for specific detection of SIV peptide inserts. Recognition of the Nef1 peptide contained within the chimeric GvpC protein was similarly demonstrated. Panel A of Figure 7 shows a Coomassie blue stained SDS PAGE gel analysis for r-GvpC^Nef1 ^presence in isolated recombinant gas vesicles or lysate of SD109 cells transformed with the pMS104D::*nef1*. Panel B of Figure 7 is a Western blot of these same samples and demonstrates that antisera from mice immunized with r-GV^Nef1 ^and also monkey anti-SHIV sera each recognize the chimeric r-GvpC^Nef1 ^protein. Thus the results demonstrate the Nef1 peptide sequence is expressed and retained by the recombinant gas vesicles used in murine immunizations. Equally important, recognition by the anti-SHIV monkey serum demonstrates that like chimeric Tat and Rev peptide containing GvpC proteins, the Nef1 chimeric protein is also perceived as containing the SIV derived peptide.

### ELISA Assessments

**Figure 8:**
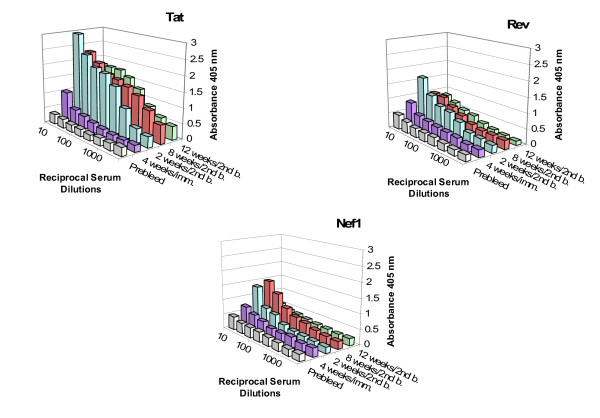
**Immunogen specific ELISA titrations of antibody elicited by rGV^Tat, Rev or Nef1 ^encoded sequences**. Mice were initially immunized using isolated r-GV and subsequently boosted as specified in the Methods. The three dimensional graphs display the temporal profiles of antigen specific antibody responses to the SIV peptide expressed within the GvpC protein. For Tat, all post-4 week samples exhibit a titer of ≥ 1:1,280 while for Rev, the maximal titer, 1:320 occurs in the week 2 post second booster sera. For Nef1, the highest titer, 1:320, is evident in sera from week 8 post second booster and by the 12 week time point, only sera from the animals immunized with r-GV expressing Rev retained a peptide specific titer.

To verify the immunogenecity of these recombinant gas vesicles and test the critical parameter, presence of the humoral immune response, mice were immunized with recombinant gas vesicles and their antibody responses to the SIV derived inserts assessed. Immunizations followed the methods and injection schedule outlined in the Methods and used isolated r-GV^Tat, Rev or Nef1^, gas vesicles produced by mutant halobacteria SD109 transformed with the plasmids pMS104D-SIV::*tat*, ::*rev*, or ::*nef1*. The serum samples drawn at various post-immunization time points were assayed by ELISA. Sera samples, fully pre-adsorbed with wt-GV as described in the Methods, were assayed to detect SIV insert specific antibody recognition by using r-GV produced by the homologous transformants and the average absorbances at 405 nm were graphed. Figure 8 presents these results in the separate three dimensional data displays and these show the sera titers at the different times during the course of immunizations. Inherently these humoral responses demonstrate the immunogenecity of each of the incorporated SIV peptides displayed by these three different r-GV. Each r-GV elicits a solid peptide specific antibody response to the initial low dose, demonstrates a secondary response to the booster and a natural waning of antibody titer over time, as shown by assay of the 12 week post booster sera. In addition, the development of an immune response inherently demonstrates both the presence of the displayed r-GvpC protein and, because the sera were fully pre-adsorbed, the immune system "visibility" of the specific SIV inserts. The titration data also hint that differences in the responses to the different inserts might not be closely linked to the actual size of SIV encoded peptide; the responses to the Tat insert (50 amino acids) was clearly more robust than the response to the Rev insert (81 amino acids). This might provide a simple and useful method to screen peptide segments that could provide an index of inherent capacity to elicit humoral responses, although additional studies would be required to demonstrate and verify ELISA based detection of inherent immunogenicity.

**Figure 9:**
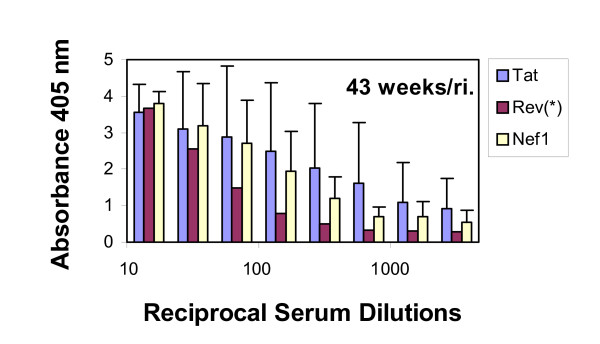
**r-GV immunizations elicit long lived antibody responses to the displayed SIV specific peptide inserts**. Seventeen weeks after the second booster, mice were boosted again with homologous r-GV and then retained for 43 weeks. ELISA assessments of sera taken at week 43 demonstrate that notable titers remain at this very late time point: 1:160 for Rev; and for Tat and Nef1, the titers are ≥ 1:1,280. **Note**: for this graph the absorbance scale is 0.0 – 5.0 and note also that (*) indicates only one animal remained in the Rev group at 43 weeks.

Features critical to immunization based protection also include the continued presence of elevated antigen specific antibody and the occurrence of such responses, independent of the specific peptide tested. Figure 9 presents ELISA titration data showing the antibody levels remaining 43 weeks after a re-immunizing booster with the homologous r-Gv. For the group immunized with r-GV^Rev ^only one animal remained at this very late time point; its serum titered at ~1:160. For the animals in the groups immunized with r-GV^Tat ^or r-GV^Nef1^, very substantial insert specific antibody titers (~1:1280 and 1:640) remained at the 43 week time point. This finding supports the conclusion that the humoral response to diverse peptides expressed using the r-GV display/delivery system can remain notably elevated. Potentially this would result from a slow release of proteins from the gas vesicle as it is degraded intracellularly and *in vitro *studies indicate this is the case (manuscript in preparation). The system therefore exhibits a characteristic desirable in an immunizing agent. In addition, the absence of exogenous adjuvant in this system provides additional evidence of the self adjuvanting characteristic of the recombinant gas vesicle delivery vehicle.

## Discussion

In a previous study we demonstrated immunologic memory for the peptide expressed by r-GV was elicited, however the assessments of r-GV in that study involved only the expression of peptide sequences from a single SIV *gag *gene [19]. The ELISA data presented here tested quite different peptide sequences incorporated into the r-GvpC protein and the results indicate that in this context, these too are stimulatory and immune visible. The results presented above extend and strengthen the initial hypothesis that r-GV can serve as a flexible cassette based epitope display/delivery system that supports expression of genes or gene segments encoding peptides from proteins representing diverse functions in their native context. Here we tested peptides encoded by three different SIVsm genes. The Tat protein can function indirectly to promote virus replication via receptor mediated signal transduction, while intracellularly Tat normally is transported into the host cell nucleus. The protein is essential for viral replication and is currently a candidate vaccine component. Rev functions as a nucleocytoplasmic shuttling protein normally found primarily in host cell nuclei/nucleoli. One of its functions is to "escort" unspliced viral mRNA out of the nucleus. In contrast, Nef is a peripheral membrane protein, and appears to have a linker function, among others. Thus Nef mediates a variety of protein-protein interactions via its SH3 binding surface and down regulates MHC class I, and CD4 expression, perhaps through its capacity as a linker. Nef therefore can exert significant effects on the host cell functions [21-27]. From the data presented here, clearly the DNA segments tested, representing sequences from proteins with significant inherent functional differences, were stably incorporated into the *gvpC *gene. The data also shows the insert peptide is retained in the recombinant GvpC protein, and the chimeric proteins produced are effectively incorporated to produce recombinant gas vesicles. For all three test peptide sequences the functionality of the r-GvpC was shown by the generation of gas vesicles that float, and r-GvpC release from isolated gas vesicles during sample preparation for SDS-PAGE, verifies r-GvpC were incorporation as part of the organelles themselves. Likewise, immunogenicity was demonstrated through the anti-peptide responses elicited. These demonstrated r-GV effectiveness as a delivery vehicle and support the conclusion that irrespective of the gene source, the displayed peptides are immune system visible and immuno-stimulatory. The known structure of GV microparticles and their resistance to degradation, support the suggestion that these functional characteristics of this unusual display system are keys to their effectiveness as an adjuvant.

The basis of this self adjuvanting effect remains to be clarified but characteristics of certain halobacterial proteins may be relevant. Molecular chaperones are essential components of organisms that function as part of the normal physiology, and in response to stress, and pathology. A molecular chaperone system also is present in *Halobacterium sp*. NRC-1 and represented by the proteins DnaK, DnaJ, GrpE, and Hsps1–4 (small heat shock proteins). The peptide-binding function of Hsp's (heat shock proteins) allows chaperon proteins such as Hsp70 (heat-shock protein 70) to acquire antigenic proteins within cells, even when administered outside the cell and thereby induce priming of CD8^+^T lymphocytes *in vivo*. In addition to peptide binding and delivery, Hsp70 also can assist as a carrier of peptides or proteins that are effective immunogens for B cells, CD4^+^, and CD8^+^T cells, in the absence of an adjuvant. Thus it has been shown that the C-terminal portion of Hsp70 (amino acids 359–610) induces human monocytes to produce CC chemokines (chemotactic cytokines), IL-12 (interleukin-12), TNF a (tumour necrosis factor alpha), as well as stimulating the maturation of dendritic cells [28]. Additionally there is evidence that for microbial Hsp, this C-terminal portion of Hsp70 is involved as an adjuvant in the induction of the innate and adaptive elements of the immune response [29].

The slow degradation of wt- and r-GV could readily initiate Hsps up-regulation in the host cell. Therefore, the self-adjuvanting characteristics of gas vesicles could be analogous to the chaperon activities of Hsp 70, produced by the host cells in response to perturbation by the gas vesicle presence, and in fact there is a notable similarity between the GvpC protein and the DnaK and DnaJ proteins. In addition, or alternatively, the geometric display on gas vesicle surfaces may be analogous to the display achieved when bacterial S-layers are used. Such displays are known to enhance immune responses [30].

Inherent features of *Halobacterium sp*. NRC-1 and their biosynthesis of gas vesicles are highly relevant to biotechnological utilities and these are evident here, in the steps to generate and test the recombinant organelles. The plasmid pFM101d allows simple insertion of exogenous DNA cassettes and subsequent transfer into the *Halobacterium *strain SD109 transformation vector. A simple restriction digest cleavage of the rightward operon is followed by ligation into pMS104d, the large (18.9 kb) plasmid used to transfer the 14 genes needed for gas vesicle biogenesis. As application of simple agarose gel analysis showed, successful SD109 transformation can be readily assessed via EtBr (Ethidium Bromide) staining to verify amplification products with the appropriate bp sizes. The actual transformation of plasmid recipient is readily achieved by a one step treatment of the Vac^- ^SD109 strain with spheroplasting solution. A cell wall, such as is characteristic of gram positive or gram negative bacteria is not present in these archaeal organisms. This fact simplifies transformation as well as the subsequent gas vesicle isolation processes. Further, spheroplast formation itself can be verified by simple microscopic examination of the treated halobacterial samples. The SD109 cells undergo a shape change and exhibit a spherical rather than the normal rod morphology (Figure 3). Such spheroplasts were readily transformed without additional treatments by simple incubation with isolated pMS104D::*tat *::*rev*:: or :: *nef1 *DNA. Similarly, successful transformation and appropriate function of the genes transferred by the transforming plasmid is also easily determined. For liquid media, the formation of "creamy-white" halobacterial cultures vs. the rust brown of Vac^- ^SD109 cultures is diagnostic for gas vesicle production and readily evident by simple visual inspection as seen in Figure 4.

The specific verifications provided here are keys in the context of validating this system and the peptides tested. First, as clearly shown, recombinant GvpC expressed by the vesicles demonstrate an expected decrease in electrophoretic mobility vis-à-vis GvpC from gas vesicles produced by wild type *Halobacterium sp*. NRC-1. Second, in the present study, the gene segments inserted into the *gvp*C encode peptides from proteins exemplifying a variety of functions, and as Figures 5, 6, 7 showed, when displayed by r-GvpC, each one was recognized not only by anti-GvpC antibody (Figure 5), validating retention of the r-GvpC protein, but also by antibody elicited *in vivo *during infection of monkeys with SHIV virus (Figures 6, 7). Since the anti-SHIV antibody did not recognize wild type GV protein (Figure 6B), the incorporated SIV peptides were being specifically identified when displayed as part of the r-GvpC protein which is present at sites all over the gas vesicle surface. Thus the insert into the GvpC protein is both immune system visible and accessible. Equally important, display of the SIV peptides in this manner did not abrogate their recognition by appropriate antisera. Finally, it is also highly relevant that as the titration of sera collected 43 weeks post re-immunization shows, titers remained for all three peptides although at 1:160, the anti-Rev titer was the lowest of the three. However, the titer for Rev likely reflects the fact that only one animal in this group was extant. At this very late time point the titers of the animals immunized with Nef1 and Tat r-GV (~1:640 and 1,280) were very notably higher.

From the perspective of general utility and ease of recombinant production, the characteristic inherent in the system used here and the effective incorporation of exogenous pathogen DNA sequences, both were demonstrated in the course of generating the recombinant gas vesicles. The features critical to their unusual utility include characteristics inherent to the gas vesicle organelle itself as well as the *Halobacteria sp*. in which they form. They are relevant to r-GV use in biotechnology applications such as a pathogen peptide display and/or delivery system. Thus, despite the unusual intracellular environment that halophiles maintain, and the unusual nature of their native proteins [31,32], the pathogen gene sequences were retained and also translated with fidelity in the halobacteria SD109 expression system as demonstrated by SHIV antibody recognition. The recombinant GvpC proteins themselves are incorporated into the gas vesicle structures as demonstrated by r-GvpC release from isolated organelles and recognition by both anti-GvpC and anti-SHIV antibody. Inherently, the GvpC protein, as the "molecular glue" for the organelle, is present at the surface of recombinant gas vesicles [12]. In addition, as previously published electron micrograph show, this surface intrinsically exhibits a highly organized, patterned surface [7,18]. Finally, gas vesicle formation itself is quality controlled by the *gvp *gene cluster carried by the transformed SD109 strain and release of the organelles themselves is easily achieved by simply suspending the halobacteria in water with 1.0 mM MgSO_4_.

## Conclusion

The studies presented here clearly demonstrate that recombinant GvpC proteins displaying peptides encoded by sequences from the three very different SIV genes each are expressed, the translated proteins are incorporated into the recombinant organelles and like native GvpC, the recombinant proteins in turn are appropriately integrated to produce functional organelles. The different chimeric r-GvpC are detected in isolated gas vesicle preparations and therefore the relevant incorporated gene sequences have been retained by transformed SD109, and the r-GvpC proteins themselves, are retained by the r-GV. The ELISA data demonstrate the r-GV, with their highly ordered surfaces serve as an effective display platform for peptide sequences as an integral part of the r-GV surface structure. Further, as shown by the ELISA data for serum samples collected at 43 weeks post re-immunization, notably elevated titers remained (Figures 8 and 9). From a practical perspective it is also relevant that the recombinant organelles also retain the natural gas vesicle functionality since they clearly float to the air/5% NaCl interface. Thus, like the wild type organelles, following halobacteria lysis by transfer to hypotonic medium r-GV can be readily isolated by centrifuge assisted flotation. Finally, from a production standpoint, it is also significant that because a standard bacterial cell wall is not present, the recombinant SD109, like the wild type *Halobacterium sp*. NRC-1, require a high salt environment (≥ 5 M NaCl) to maintain organism integrity. In the absence of high salt, the halobacteria lyse and therefore for this recombinant, display system, "escape" of viable halobacteria cannot occur. In addition as sub-cellular organelles the r-GV, unlike the various permutations of bacterial ghosts [33,34], contain only a very limited number of "foreign" proteins. Therefore in this they differ significantly from the complex array of protein and carbohydrate inherently present in the bacterial ghost based delivery systems. Taken together, from the perspective of cost and recombinant containment, and extended retention of elevated humoral immune response elicited, the submicroscopic particles that constitute the halobacterial gas vesicle system have the potential to provide an alternative antigen display and delivery system. The ease of tailoring specific epitope presentations inherent in the surface display of peptides by the organelle would also support use of this system for displays to screen for humoral responses to specific pathogen epitopes, or as the peptide component in prime and boost immunizations [35,36].

## Methods

### Plasmid design

Plasmid pFM101d (DasSarma and Morshed, unpublished data [5,37]) carries the rightward gas vesicle operon and a unique site for insertions within *gvpC *gene that restriction enzyme Eco47 III cleaves (Figure 1); it was used in generating the three recombinant *gvpC *genes studied here. The shuttle vector containing origins of replication for *E. coli *and *Halobacterium sp*. NRC-1, and selectable drug resistance markers, as well as genes for gas vesicles (GV) synthesis [14,7] was re-constructed using plasmids pSP104, pFM101d and pFM104::*gag *504. Like pFM104d [19] this plasmid, termed vector pMS104d contains Eco47 III restriction site at the "d" location and all the genes necessary for gas vesicle synthesis. It was used in the present studies to generate the three different transformants populations each of which incorporated one of gene segments of interest, *tat *(150 bp), *rev *(243 bp) and *nef1 *(642 bp).

### Amplification of SIVsm DNA and Preparation of Plasmids

#### PCR amplification of the SIV genomic fragments

Genomic sooty mangabey monkey (SIVsmm) template DNA, a gift from Dr. V. Hirsch from NIH, was PCR amplified using standard recombinant technology methods [38]. Briefly, based on the published SIVsmm proviral genome sequence [GenBank: X14307], the primers necessary to amplify the entire genome as 21 individual fragments were designed, then synthesized commercially (Sigma-GenoSys, Woodlands TX). To restrict DNA segment sizes ≤ 650 bp, as necessary, genes were cleaved into segments and numbered accordingly. The forward and reverse primers for the fragments encoding SIV^*tat*, *rev *and *nef1 *^assessed in detail here, the relevant primers for *gvpC *amplification and the amplified DNA fragment sizes are shown in Table 1.

For each amplification, the 100 μl reaction mixture contained the following: 10 μl of Thermophilic DNA Polymerase 10× Buffer, Magnesium Free; 6 μl of Magnesium Chloride Solution, 25 mM; 2 μl of dNTP mixture (Promega Corporation, Cat. # U1330, Madison WI), 2.5 mM; 0.5 μl *Taq *DNA Polymerase, 5 Units/μl; 3 μl of template SIV DNA; 1 μl each of forward and reverse primers, 25 pmol; and 76.5 μl of ddH_2_O. The amplification program was as follows: DNA denaturation at 95°C for 2 min, followed by 34 cycles of denaturation at 95°C for 30 sec, DNA annealing at 55°C for 30 sec, and extension at 72°C for 1 min followed by sample incubation at 72°C for 5 min. The amplified DNA products were resolved electrophoretically on agarose gels and visualized by ethidium bromide staining.

#### Plasmid Preparation and Amplifications

As described in detail previously [19], the generation of transformed Vac^- ^(minus) halobacterial SD109 mutants used a series of plasmids. The initial PCR products provided SIV fragments and following phosphorylation, the selected SIV fragments and the pUC19 vector (New England BioLabs, Beverly, MA), cleaved with Sma I, were dephosphorylated using Shrimp Alkaline Phosphatase (SAP, Roche Diagnostics Corporation, Indianapolis IN). Fragments were gel purified and separately ligated with T4 ligase (Amersham Pharmacia Biotech, Inc; Piscataway, NJ). *E. coli *cells (DH5α) were rendered competent using the standard treatment with calcium chloride as detailed previously [38,14], then transformed and the incorporated SIV DNA amplified. Each SIV containing insert was subsequently removed by digesting the isolated plasmid with Sma I. Vector pFM101d was cut with Eco47 III, dephosphorylated using SAP, gel purified and subsequently the SIV inserts were blunt-end ligated into the Eco47 III site of the *gvpC *gene contained in the plasmid pFM101d shown in Figure 1. Aliquots of competent DH5α cells (Invitrogen, Carlsbad, CA) were then transformed with plasmids termed pFM101D::*tat*,::*rev*, and ::*nef1 *and grown in Luria broth (Fisher Scientific, Pittsburg, PA), containing 20 mM glucose and 100 μg/ml Ampicillin (Sigma-Aldrich, St. Louis, MO), the selectable marker for amplification in *E. coli*. Using the published DNA sequences for *gvpC *and for each SIV fragment, plasmid DNA was assessed by PCR amplification to verify the presence of SIV^*tat*, *rev *or *nef1 *^insertion using a *gvp*C forward primer and the reverse primers for the respective *tat, rev *or *nef1 *gene segments (Table 1). All plasmid constructs were routinely characterized by restriction enzyme analysis and after confirmation that the plasmid DNA included *gvp*C containing the appropriate insert and orientation; they were excised from pFM101D using Spe I and AsiS I (Figure 1). Following gel purification, the fragment with the rightward operon containing the *tat, rev *or *nef1 *insert was ligated into pMS104d, also cut with Spe I and AsiS I. The resultant insert containing plasmids, pMS104D::*tat*, ::*rev *and ::*nef1 *were verified by restriction enzyme digestion and/or PCR analysis, then the plasmid construct with its SIV DNA cassette insert was sequenced using an ABI sequencer to verify the SIVsm inserts to confirm that no deletions or mutations had occurred. Like pFM104d [19], this plasmid, pMS104d is 18.9 Kb in size and contains the origin of replication for halobacteria and the selectable marker, mevinolin. Restriction enzymes were obtained from New England Biolabs (Beverly, MA), DNA polymerase from Promega (Madison, WI) and T4 DNA ligase was included in the SureClone™ Ligation Kit (Amersham Pharmacia Biotech, Inc; Piscataway, NJ); each component was used as specified by the manufacturer. For final validation, cloned DNA or PCR amplified SIV fragments were sequenced using an ABI 377XL automated DNA sequencer at the Sequencing Facility at the University of Massachusetts, Amherst or an ABI 3730 sequencer at the Microbiology Department, in conjunction with the ABI PRISM Big Dye Terminator v.3.1 cycle sequencing kit. Following sequence confirmation, the derived SIV sequence containing plasmids, pMS104D::*tat*,:: rev and ::*nef1 *were used to transform the Vac^- ^(minus) halobacterial strain SD109.

### Growth and transformation of gas vesicle deficient Halobacterium sp. strain SD109

SD109, a mutant strain of *Halobacterium sp*. NRC-1 lacking the gas vesicle gene cluster, is deficient in gas vesicle formation. Using a modified protocol for transformation of *Halobacterium sp*., recombinant plasmids each with one of the SIVsm gene segment, were transformed into the Vac^- ^SD109. The methods used here are those optimized for *Halobacterium salinarium *[6] and are described in detail in the Archaea: A Laboratory Manual, Protocol 29 and Appendix 2 [39]. Colonies were scored visually for the gas vesicle phenotype (Vac^+^) and Vac^+ ^colonies expanded (Figure 4), using high salt peptone (Oxoid Ltd, Basingstoke, Hampshire, UK) with 10 μg/ml mevinolin as described previously to select for the recombinants [18,19].

### Expansion of SD109 Transformant populations and gas vesicle isolation

Transformant populations were expanded using growth at 37°C on agar plates containing added trace metals (ZnSO_4_, MnSO_4_, CuSO_4 _and FeSO_4_) and the selective antibiotic, mevinolin (CM+Mev+). Several colonies were then selected from each transformation and grown in liquid media under the same selective conditions. The retention of the inserted SIV DNA fragments was verified by restriction analysis and validated transformant cultures were grown at 37°C using larger plates to produce recombinant populations from which gas vesicles would be harvested. Gas vesicles from these transformed SD109 were harvested by cell lysis using water containing 1.0 mM magnesium sulfate and subsequently subjected to 3–5 rounds of centrifugally assisted flotation to isolate the organelles from cell debris as described previously [18,19]. Gas vesicle yields were first assessed spectrophotometrically (OD_596_) and then precise concentrations were determined using the Biorad protein assay (BioRad Laboratories, Hercules, CA). Isolated gas vesicles were stored in 5% NaCl at 4°C.

### Gas Vesicle Analyses

#### SDS-PAGE

Aliquots of isolated wt-GV and recombinant gas vesicles (r-GV^Tat, Rev, or Nef1^), dialyzed against distilled water, were mixed with 5xSDS-PAGE sample buffer (Pierce-Endrogen, Rockford, IL), and heated 5 min in a boiling water bath. Then, 20–25 μl were loaded into wells of Novex pre-cast 8–16% or 12% gradient gels (Invitrogen, Carlsbad, CA) and electrophoresed until the tracking dye reached the bottom of the gel. Gels were stained with Coomassie Blue R250 (Sigma-Aldrich, St. Louis, MO) or electroblotted for Western analysis. MultiMark standards (Invitrogen, Carlsbad, CA) and SeeBlue PreStained Standards (Invitrogen, Carlsbad, CA) were used to assess the molecular weight (MW) of protein species.

#### Western blot

For Western blots, proteins were transferred from the SDS-PAGE gel to Immobilon-P NC membrane or PVDF membrane (Millipore, Bedford, MA) using a BioRad transfer apparatus (BioRad, Hercules, PA) following the manufacturer's instructions. Subsequently, membranes were blocked with 5% non-fat dry milk (NFDM) in phosphate buffered saline (PBS; pH 7.2). This and all other incubations were carried out at RT on an orbital shaker. After blocking for 2.5 h, membranes were rinsed three times with 1% BSA/PBS, and then probed by incubation with immune serum, or the appropriate control serum. GvpC protein was detected using 0.020 μg/μl rabbit anti-GvpC antibody diluted in 1% BSA/PBS. SIV peptides expressed as part of the recombinant gas vesicle r-GvpC protein were identified with monkey anti-SIV sera (RHE 544, a gift from Dr. V. Hirsh, NIH). The SHIV-infected macaque plasma (SHIV variant 89.6PD from the animal with number R94085) was a gift from Dr. D. Pauza, Institute of Human Virology, Baltimore, MD) [40]. These virus specific sera were diluted 1:150 or 1:200 respectively using 1% BSA/PBS; mouse anti-Nef1 sera were used at a 1:250 dilution. Following 1.5–2 h incubation with anti-sera (SIV or SHIV), membranes were rinsed three times in 1% BSA/PBS. Specifically bound antibody was detected by incubation with either alkaline phosphatase conjugated goat anti-rabbit IgG (H+L) chain (Jackson Immuno Research, West Grove, PA) or with alkaline phosphatase conjugated rabbit anti-monkey antibody (Sigma-Aldrich, St. Louis, MO). Each conjugate was diluted 1:1,000 in 1% BSA/PBS and incubation continued for 1 hour. Membranes were rinsed three times with 1% BSA/PBS and two times with dH_2_O, then incubated with the alkaline phosphatase membrane substrate, SigmaFast (Sigma-Aldrich, St. Louis MO); protein bands developed within 5 min of substrate addition. Subsequently blots were rinsed in distilled water; air dried at RT and archived as scanned images.

#### Antigen preparation and immunizations

Mouse anti-SIV sera raised against protein encoded by SIV DNA insert sequences was generated by immunizing BALB/c mice (Jackson Laboratories, Bar Harbor, ME) with subcutaneous injections of recombinant GV in sterile PBS as outlined in the IACUC protocol specifically approved for these studies. Sera from immunizations with the expressed SIV inserts of Tat, Rev or Nef1 derived from gene segments inserted into the *gvpC *gene. Sera were collected from mice by tail bleed and the pre-bleed sera from these same mice were pooled and used as a matched control serum.

Briefly, to test immunogenecity, 4 month-old mice were each injected subcutaneously at multiple sites on the upper back initially using low dose immunization (1–3 μg total protein) with recombinant gas vesicles (r-GV^Tat, Rev, or Nef1^) in 200 μl sterile PBS. The boosters used 50 μg total proteins per mouse; delivered in the same manner [41]. Sera were collected by tail bleed 2 and 4 weeks after primary immunization and at 4 weeks following the first booster. After the second booster, sera were collected at 2, 4, 8, 12 and 17 weeks. Seventeen weeks after the 2^nd ^booster the mice were re-immunized using the same protein concentration and sera were collected 10 days later. The animals were then retained for a total of 43 weeks post-re-immunization and their sera were collected at that final time point. Sera were subsequently adsorbed and assayed by ELISA as described below.

### Adsorption of immune sera and ELISA assays

Prior to ELISA antibody titration assays, immune sera were adsorbed to remove any antibody directed against the surface exposed wt-GvpC protein by incubations with wt-GV. After serum adsorptions, the gas vesicles were removed by 14,000 RPM centrifugation at room temperature (RT) which collapses the organelles, and the overlying serum was aspirated. Removal of anti-GV antibody was verified by standard ELISA using wt-GV as antigen. The test antigen used deflated (collapsed) gas vesicles prepared as follows. Isolated gas vesicles produced by wt-NRC1 or SD109 transformants were separately isolated, then deflated by brief micro centrifuge centrifugation; this drives the gas from the organelles yet retains the gas vesicle intact. Deflated gas vesicles were diluted to 1 μg per 100 μl in PBS, (pH 7.2) and served as the specific SIV^Tat, Rev or Nef1 ^peptide antigen. ELISA wells (Immobilon, Fisher Scientific, Pittsburgh, PA) were filled with 100 μl of the appropriate wt- or r-GV^Tat, Rev, or Nef1^/PBS solution, and then incubated for 1 hr at RT. After three rinses with PBS, wells were blocked for 1 hr at RT with 200 μl per well of 3% BSA/PBS (Sigma-Aldrich, St. Louis, MO). Wells were again rinsed with 200 μl PBS. Adsorbed immune mouse sera or pre-bleed sera were serially diluted in 0.1% BSA/PBS. Wells were filled with 100 μl of diluted sera and incubated for 1 hr at RT, then rinsed 4 times with 200 μl of 0.1% BSA/PBS. Bound mouse immunoglobulin was detected using alkaline phosphatase conjugated affinity purified goat anti-mouse IgG (H+L) (Jackson Immuno Research, West Grove, PA), diluted 1:1,000 in 0.1% BSA/PBS; 100 μl was added to each well. Following 1 hr incubation at RT, wells were rinsed five times with 0.1% BSA/PBS then each well received 100 μl of pNPP substrate. The pH 9.8 substrate contained distilled water with 10% diethanolamine, 0.5 mM MgCl_2_, as a stabilizer (Fisher Scientific, Pittsburgh, PA) and 1 mg/ml of Sigma 104 para-nitrophenylphosphate (Sigma-Aldrich, St. Louis, MO). Following an incubation of 60 or 90 min at 37°C, the absorbance at 405 nm was quantified using a VersaMax ELISA Reader (Molecular Devices, Palo Alto, CA). All samples were assayed in triplicate.

## Abbreviations

wt-GV, wild type gas vesicles; r-GV, recombinant gas vesicles; Gvp, gas vesicle protein; SIV, Simian Immunodeficiency Virus; SIVsm, Simian Immunodeficiency Virus infecting the sooty mangabeys; SHIV, Simian-Human Immunodeficiency Virus; Å, Angstrom; bp, base pair; BSA, Bovine Serum Albumin; EtBr, Ethidium Bromide; dNTP, deoxynucleoside triphosphates; nm, nanometers; NC, Nitrocellulose (membrane); OD, optical density; pI, isoelectric points; PBS, Phosphate-Buffered Saline; PCR, polymerase chain reaction; PVDF, Polyvinylidene fluoride (membrane); RT, room temperature; SDS, sodium dodecyl sulphate.

## Authors' contributions

This work was a portion of doctoral studies carried out in partial fulfilment of the requirements for a Ph.D in Microbiology at the University of Massachusetts (MS). Both authors contributed substantially to the work and manuscript presented here. As senior author and PI of the study, ESS developed the plan and supervised the work, and finalized the manuscript.
